# Clozapine rechallenge and initiation despite neutropenia- a practical, step-by-step guide

**DOI:** 10.1186/s12888-020-02592-2

**Published:** 2020-06-05

**Authors:** Edward Silva, Melanie Higgins, Barbara Hammer, Paul Stephenson

**Affiliations:** 1 Consultant Forensic Psychiatrist, Ashworth Hospital, Mersey Care NHS Foundation Trust, Parkbourn, Maghull, Merseyside, L31 1HW UK; 2 Consultant Haematologist, Arrowe Park Hospital, Arrowe Park Road, Upton, Merseyside, Wirral CH49 5PE UK

**Keywords:** Agranulocytosis/drug therapy*, Antipsychotic Agents/administration & dosage/*adverse effects., Clozapine., Clozapine/*adverse effects., Forensic mental health services., Granulocyte-Colony-Stimulating Factor/*therapeutic use., Lithium/*therapeutic use., Neutropenia/blood/chemically induced/ethnology/*therapy., *Neutrophils., Schizophrenia/drug therapy*.

## Abstract

Clozapine remains the only drug treatment likely to benefit patients with treatment resistant schizophrenia. Its use is complicated by an increased risk of neutropenia and so there are stringent monitoring requirements and restrictions in those with previous neutropenia from any cause or from clozapine in particular. Despite these difficulties clozapine may yet be used following neutropenia, albeit with caution. Having had involvement with 14 cases of clozapine use in these circumstances we set out our approach to the assessment of risks and benefits, risk mitigation and monitoring with a practical guide.

## Background

### Authors’ experience

This guide is based upon the authors’ experience since 2006 of treating patients with clozapine following neutropenia in a UK high secure forensic hospital. This patient group presented combinations of treatment resistant schizophrenia, severe behavioural disturbances requiring the use of extended seclusion in addition to contraindications to the use of clozapine secondary to clozapine induced or other neutropenias. Given the lack of effective alternatives and the unacceptable clinical condition of the patients we began to re/use clozapine with an evolving approach to risk assessment, monitoring and mitigation. The results were very much better than expected. Clozapine was re/introduced to 14 patients despite neutropenia due to a variety of aetiologies; benign ethnic neutropenia (BEN), neutropenias secondary to both clozapine and other drugs and autoimmune neutropenia. Despite previous prolonged periods of detention in conditions of high security, half of our group improved sufficiently to transfer out of high security. There were significant reductions in the use of seclusion and there were no serious adverse events.

## Main text

### General warning

Any team considering clozapine in a patient with previous blood dyscrasia should proceed with great caution. All approaches are hazardous. The existing best evidence is derived from a small number of nationwide pharmacovigilance studies reporting crude outcomes on clozapine rechallenge in combination with individual case reports and series that will inevitably be affected by publication bias. These have been subject to meta-analysis [1]. The evidence relating to the use of drugs to support neutrophil counts is derived from case reports and series only. Decision making is entirely based on the individual circumstances of the patient, in the absence of any agreed protocols and with uncertainty regarding risks. Despite shared decision making having some advantages, we have found it helpful for responsibility regarding final decisions resting clearly with the consultant psychiatrist prescribing clozapine. Although treatment and mitigation planning itself is an extremely useful process, the plans developed will invariably have to be modified in the light of the patient’s response and local team and resources issues; changes to treatment and neutropenia management is often required quickly. It is very helpful to acknowledge this in the plan itself. We have also had experience of the use of enforced naso-gastric clozapine [2] and with clozapine given after severe physical health comorbidities [3]; whilst clear and informed clinical decision making is essential, careful thought must also be given to the team and institutional factors that can facilitate or interfere with these sorts of intrusive and unusual interventions [4].

### Clozapine and neutropenia

Clozapine continues to have unique efficacy in treatment resistant schizophrenia but remains a third line treatment on account of the increased risks of neutropenia and agranulocytosis [5, 6]. Whilst the initial indications arose from a series of deaths in Finland [7, 8] the subsequent risk monitoring and management regulations have resulted in deaths from these adverse effects being extremely rare. The rates of clozapine induced neutropenia (CIN) and agranulocytosis (CIA) are 3.8 and 0.9% but the incidence of death secondary to these complications, as managed by the monitoring arrangements, is 0.013 and 2.1% respectively [9]. The UK national pharmacovigilance has identified eight clozapine related blood dyscrasia deaths to date [10]. International regulations for clozapine monitoring vary [11]. The UK clozapine blood monitoring requirements are summarised in Table 1. In the UK, clozapine may only be used within licence as a third line treatment if there is no previous history of neutropenia from any cause and with blood monitoring.

**Table 1:** UK Clozapine Monitoring and Risk Management Requirements

Standard		
Colour alert	WBC × 10^9^/L	Neutrophils × 10^9^/L
Green	> 3.5	> 2.0
Amber	3.0–3.5	1.5–2.0
Red	< 3.0	< 1.5
**Benign Ethnic Neutropenia**		
**Colour alert**	**WBC × 10**^**9**^**/L**	**Neutrophils × 10**^**9**^**/L**
Green	> 3.0	> 1.5
Amber	2.5–3.0	1.0–1.5
Red	< 2.5	< 1.0

Neutrophil counts are categorised according to a colour-coded system [10]; if a patient has an Absolute Neutrophil count (ANC) <  1.5 ×  10^9^/L (red alert) clozapine treatment must be stopped immediately. A lower threshold is available for patients with a heamatologicaly confirmed diagnosis of Benign Ethnic Neutropenia (BEN). The UK requirements have not yet aligned to use the lower neutrophil thresholds now used in the United States [12]. Afternoon blood sampling may optimise clozapine blood testing, ideally after exercise; this alone has been demonstrated to be sufficient to improve outcomes [13]. CIN and CIA are idiosyncratic, unpredictable type B adverse drug reactions [14, 15]. The hypothesis of an immune mediated mechanism is supported by the timing of initial adverse effects and the tendency to have faster and more severe problems on rechallenge [16]. Genetic linkage studies suggest both immune and anion transporter gene related mechanisms [17–20] but are not sufficiently predictive to be clinically useful [21]. The metabolites of clozapine may be toxic to neutrophils [22].

### Clozapine Rechallenge after CIN and CIA

The best available evidence regarding repeat dyscrasias with clozapine is conflicting. A 2006 publication of all available clozapine rechallenges in the UK and Ireland found the majority did not experience a repeat dyscrasia: 38% of patients (*n* = 20) experienced a repeat dyscrasia with a significant increase in severity, duration and speed of onset. However there were no deaths; all repeat dyscrasias resolved when clozapine was discontinued. The majority of dyscrasias occurred within 18 weeks of clozapine treatment; four occurred after this [23]. In 2016 data from a nationwide pharmacovigilance study in Argentina presented the outcomes of 19 patients rechallenged with clozapine; approximately one third of patients experienced a second dyscrasia which mostly occurred sooner during treatment but were less severe and of shorter duration [24]. Evidence from case reports shows a positive outcome in more than 60% of clozapine rechallenges after neutropenia (128 positive outcomes from 203 cases); there is less reported success following agranulocytosis (3 positive outcomes from 17 cases) [1].

### Risk-benefit analysis

The risk-benefit analysis includes the likely benefit in terms of reduction of psychiatric morbidity following the use of clozapine balanced against the risks of a repeat dyscrasia, the risks of death or other adverse outcome secondary to a dyscrasia and the possible harms from the risk mitigation strategies used.

### Benefits

1. The indication for clozapine challenge / rechallenge:
  Precisely define the current indication for clozapine and the supporting evidence. If clozapine has been used previously, was the clinical response sufficient to justify a rechallenge? If insufficient, were there problems related to the duration or dose / level achieved? If clozapine was previously discontinued, then is the response to the current treatment inadequate? Alternatives may have been attempted, although these are highly unlikely to be effective [25]. Given the risk and uncertainty of clozapine rechallenge and advice against the routine use of lithium or Granulocyte Colony Stimulating Factor (G-CSF) [26], the severity of the patient’s condition should be at least severe if not extreme [27]. Very significant resistant psychotic symptoms should contribute to markedly impaired function, prolonged detention or serious risk to self or others.
2. Likelihood of benefit from clozapine: Patients who either had a previous good response to clozapine then dramatic deteriorations on withdrawal or who have never had clozapine present the groups most likely to benefit. Clozapine naïve patients have an approximately 33–40% chance of significant improvement [28, 29]. Patients who have had limited previous clinical improvement with clozapine present uncertainties; some will have had partial responses and the records may not allow a full assessment. Most who make a good response to clozapine, as with other antipsychotics [30] do so relatively quickly, with noticeable improvements within weeks of initiation. An adequate trial of clozapine should consist of at least 8 weeks of therapy with plasma trough level above 350–400 mcg/L; some young men, particularly smokers, may require doses in excess of 900 mg/d to achieve this [31]. However, some may respond much later in treatment, although frequently sub-optimally, but even a small improvement may be sufficient to improve quality of life and function in groups as severely affected and restricted as ours [32, 33]. In these cases there may remain the possibility of sufficient improvement to achieve management of violence [34] and or discontinuation of seclusion [2] if not full symptom control. Rechallenge should not be discounted in cases where the previous response was less than good, relatively brief or when plasma levels indicate room for dose escalation [31, 33, 35]; however, there will be increased uncertainty. Patients who discontinued clozapine after lengthy periods at substantial doses with higher than usual plasma levels without a response resulting in significant clinical improvements should not be rechallenged.

### Risk assessment and information gathering

1. Is clozapine really prohibited?
  The UK license for clozapine has changed. Some will have had clozapine withdrawn on a single red result only, with unrecognized Benign Ethnic Neutropenia (BEN) [36]or on the basis of one or more “amber” results. A search for the contemporaneous notes may be required in addition to liaison with the previous clozapine provider. Resolving this may allow the reuse of clozapine on license with no additional safeguards.
2. Is another neutropenia suggested?
  - BEN: In our practice defining ethnicity may be complex and so a family history may be required to determine if a patent’s ancestry includes affected groups. In those who are members of an affected ethnicity obtain all available pre-clozapine blood tests, full blood counts (FBC), and look for previous results of low neutrophil counts in the absence of infection or other pathology. If found, liaise with a haematologist having performed some baseline blood tests [37] asking a specific and relevant question: “*Mr X is a Y year old of E ethnicity with no history of spontaneous infection or other serious physical illness. Repeated FBCs have shown neutrophil counts both above and below the normal reference range. Other tests including a blood film, antibody screening, myeloma screen, CRP and haematinics are normal. The abdominal ultrasound does not show splenomegaly. Is this consistent with a diagnosis of BEN and can you confirm this to allow registration with the clozapine monitoring service using the BEN reference ranges?”* However, a number of patients with BEN may also have pre-treatment neutrophil counts too low to allow the use of clozapine within license. BEN has recently been shown to have a genetic origin, with all cases of BEN having the Duffy-null genotype [36]. This can be determined with an extended blood type, but not all those with the Duffy-null genotype will have BEN. This finding may be highly significant given the finding that British patients of African ancestry with this genotype have an approximately 20 fold increased risk of a neutropenia with clozapine; however, this is most likely to be benign [20, 38].
  - Viral illness: It may be possible to demonstrate a temporal relationship between low neutrophil counts and a viral illness. However, this requires caution given that initial adverse effects of clozapine commonly include fatigue, fever and a headache [16].
  - Other drug induced neutropenia: the initiation of one of the other implicated drugs may be the cause of neutropenia, particularly if this occurs after 18 weeks or preferably one year of clozapine treatment. Implicated drugs include; carbimazole, sodium valproate, lamotrigine, mianserin, mirtazapine and carbamazepine [39, 40]. For cases with low neutrophil counts prior to clozapine, stopping implicated drugs may result in neutrophil counts improving to the extent that further interventions are not required although clozapine would still be used off license.
3. In the event of previous clozapine induced neutropenia (CIN) or agranulocytosois (CIA): how severe was the previous blood dyscrasia? What was the speed of onset, nadir, duration and presence or otherwise of infection? Whilst reports are encouraging in rechallenge following CIN, the use of clozapine after CIA is less likely to be successful and carries significantly more risk of a life threatening complication.
4. Possible clozapine rebound psychosis
  Paradoxically, restarting clozapine for some of our patients brought both potential risks and benefits to their mental states on account of clozapine withdrawal rebound psychoses even more extreme than their baseline [41]. This lends more weight to a more assertive approach to supporting neutrophil counts as well as enforcing clozapine [42] to avoid the most extreme disturbances.

### Attempt risk stratification for repeat dyscrasia

#### Making a determination of the relative risk of a blood dyscrasia will help determine which strategy to use

1. No known / minimal increased risk: clozapine stopped prematurely on account of amber results, unconfirmed red results or stopped / not used in previously unrecognised BEN.
2. Possible increased risk: BEN with low pre-treatment neutrophil counts.
3. Some increased risk: previous non-clozapine drug related blood dyscrasia not occurring during clozapine therapy.
4. Uncertain risk: neutropenia during clozapine therapy occurring later in treatment following the introduction of another drug implicated in neutropenia. This will, to an extent, depend on the risk associated with the drug in question. Carbimazole is for example associated with a greater risk of neutropenia than clozapine. Clozapine may share an aetiology for neutropenia with other drugs via human leukocyte antigen (HLA) related mechanisms [43]
5. Probably increased risk: neutropenia during clozapine therapy early in treatment following the introduction of another implicated drug.
6. Clozapine related neutropenia:
  - Increased risk: previous CIN. Take into account the speed of onset, duration and time to recovery [23, 24].
  - Significant increased risk: CIA with no infection [9].
  - High risk: CIA plus infection.

### Risk stratification related to management if clozapine withdrawn

1. Previous clozapine discontinuation without serious problem behaviour.
2. No previous clozapine treatment. No serious problem behaviors following withdrawal of previous non-clozapine antipsychotics.
3. Known rebound psychosis on withdrawing clozapine without serious problem behaviours.
4. Rebound psychosis on clozapine withdrawal resulting in moderate problem behaviours.
5. Rebound psychosis on clozapine withdrawal plus life threatening problem behaviours.

## Conclusions

### A stratified approach to clozapine rechallenge after CIN / CIA and initiation after other neutropenias

1. Clozapine alone

There are a number of circumstances in which clozapine alone might be used in the face of previous reports of neutropenia:

- The simplest cases: misreported ‘red alerts’ either unconfirmed or not below threshold for previously undiagnosed BEN allow registration and use of clozapine within license without additional safeguards.
- Neutropenia due to other drugs historical or ongoing: if stopping another implicated drug results in a rapid increase in neutrophil count then there is a good case to be made to initiate clozapine with an off-license agreement. A team may wish to have a contingency plan in the event of a further episode of neutropenia. The contingency plan should reflect the severity of the patient’s dyscrasia and psychosis.
- Low pre-treatment neutrophil counts with or without BEN: optimise blood sampling. Changing sampling time from morning to afternoon (ideally after exercise) may be sufficient to allow initiation and on-going treatment [44–47]. Longer-term processes are necessary to ensure this continues. Alternatively, following consultation with the clozapine monitoring service and a haematologist alongside an assessment of the risk of clozapine withdrawal and the degree of patient compliance, arrangements can be made for accepting lower neutrophil count thresholds before clozapine must be stopped: 1.0 × 10^9^/L or lower. In the United States these thresholds have recently been lowered [12]. In an oncology setting, action is not taken for absolute neutrophil counts below 0.5 × 10^9^/L unless there is evidence of infection [48]; however, in the mental health setting, risks are associated with this approach since patient cooperation may be minimal and the difficulties of managing an uncooperative physically sick patient extreme.
- BEN with treatment emergent recurrent sub-threshold neutrophil counts without neutropenia: an advantage of the neutrophil monitoring requirements is that they include a large margin of safety. A disadvantage is that for some patients with BEN there may be repeated sub-threshold results (ANC between 1.0–1.5 × 10^9^/L) without sepsis and a requirement for multiple blood testing. If a patient will cooperate with this then there is no difficulty. It may be necessary to consider an agreement to continue to use clozapine but continue sampling at weekly or fortnightly intervals if the patient presentation justifies this. Identification of the Duffy Null genotype (the CC genotype of the rs2814778 variant of ACKR1) in those with African ancestry shows a robust association with low neutrophil counts and is thought to be the cause of BEN, thus allowing a more definitive diagnosis rather than the current "diagnosis of exclusion". This population has a more than 20 times increased risk of neutropenia on clozapine, most often as a result of BEN rather than an adverse drug reaction [20].
- Drug treatments to support neutrophil counts

If neutrophil counts cannot be sustained at adequate levels, the use of lithium or Granulocyte Colony Stimulating Factor (G-CSF) can be considered if the risk of a dyscrasia and managing a rebound psychosis is considered too great. Tables 2 and 3 outline the use of G-CSF and Lithium. Our approach has evolved. Our first case involved a patient who had had multiple episodes of non-clozapine drug included neutropenia with sepsis, CIN with severe clozapine withdrawal psychosis and prolonged hospitalization, extensive violence, self-harm and extremely long seclusion; prior to clozapine withdrawal there had been an excellent clinical response. The risks of rechallenge were high; the haematology advice favoured G-CSF over lithium to be used from the outset. In less extreme cases lithium can be a useful first line as set out by Manu [72].

- *BEN followed by treatment emergent CIN*:
  Lithium or G-CSF can be used to support neutrophil counts depending on the severity of the neutropenia and the patient’s history when psychotic. Lithium or G-CSF may be used to continue clozapine treatment [53, 76, 77]. Lithium or G-CSF can be given without interruption of clozapine treatment. Decision making will balance the risks of clozapine withdrawal against those of a dyscrasia and the likely benefit of the strategy used. The initial approach we used for one patient, with the assistance of a haematologist, was to give G-CSF as required (plus-rescue). After each dose of G-CSF there was an excellent response in neutrophil count; however, after eight episodes of neutropenia with a nadir of 0.4 × 10^9^/L the patient was established on regular G-CSF. Although clozapine naïve, this patient had had several episodes of life threatening self-harm and of serious assaults on others in the context of active psychosis.
- *CIN with gradual deterioration on clozapine withdrawal, disturbed behaviour (not the most extreme)*:
  Lithium appears to have a good record of success when used to support clozapine rechallenge and has fewer difficulties in terms of side effects, administration, cost and downstream issues. There is little evidence regarding dose but in our experience a serum level of greater than 0.4 mmol/L has been sufficient to have a meaningful effect on neutrophil counts. This is easily assessed for each individual patient. If required, lithium can be replaced with G-CSF. These approaches were all successful in our patient group.
- *CIN with rapid deterioration on clozapine withdrawal, disturbed behavior (extreme)*:
  These patients present by far the most extreme problems in terms of the severity of neutropenia and psychosis. We have used prophylactic G-CSF in this group, with haematology assessment and support regarding dose, interval and monitoring.

3. *For all cases*

- *Avoid other implicated drugs.* For all patients we avoid the use of any other drugs implicated in neutropenia. This is most relevant if there is a history of clozapine induced seizures or if affective symptoms are prominent in which case topiramate [78] may be preferable to valproate, lamotrigine or carbamazepine.
- *Planning and Liaison.* Careful teamwork will be required. A variety of simple practical steps were taken to ensure that G-CSF was available on the wards where it might be needed, with simple plans for its use should this be required by on-call junior medical staff. Whilst we increased the frequency of FBC testing (arbitrarily to initially twice weekly) plans were made to ensure that this was optimized in the afternoons, but not so late that results only became available out of hours. Liaison with other medical professionals can sometimes be confused with attempts to diffuse responsibility. It will be the treating psychiatrist who is responsible for the decision-making and not the haematologist. On occasion haematologists report requests from psychiatrists asking whether or not clozapine should or should not be restarted, rather than to advise on the hematological risks and how these might be managed. For cases when capacity is impaired or when patients do not consent, the difficulties and uncertainties must be shared with the legal power authorising treatment. The use of medication to support rechallenge may need to be specified depending on the approach taken by the approving body. In all cases, off-license agreements will be required with the clozapine provider. Family liaison will be important. In several of our cases both patients and their families, recognizing the benefits when clozapine had been used previously and the severity of the deterioration when it was withdrawn, were eager to rechallenge.
- *Retitration rates.* Given the probable contribution of an immune mechanism, a slower clozapine titration may be useful; as yet there is no evidence to support this [79].
- *How long to continue Lithium or G-CSF.* This is not known.
- *Timing.* Clozapine rechallenge is anxiety provoking. Given the types of judgments required, we planned our blood sampling regimes to avoid results out of hours or when key decision makers were away from the hospital.

4. ***Downstream***

**Table 2:** G-CSF

Physiological role	cytokine glycoprotein stimulating differentiation, release and survival of neutrophils and other granulocytes
Drug	Filgrastim (recombinant G-CSF) has been available since the early 1990’s; originally to treat patients with chemotherapy-induced neutropenia
Current usage	Severe chronic neutropenia, mobilisation of haematopoietic progenitors for stem cell transplantation [49–51], drug induced neutropenia [52].
G-CSF and clozapine rechallenge after CIN / CIA	Multiple case reports [49, 53–65] reviewed by Lally et al [66, 67]; G-CSF successfully supported over 70% of initial CIN cases. Success was markedly reduced following CIA.
Common adverse effects and management	Flu like symptoms, bone pain, headache, pyrexia and fatigue.
Rare adverse effects	Splenic rupture, glomerulonephritis, alveolar haemorrhage, thrombocytopenia and capillary leak syndrome.
Side effect management and monitoring	Analgesia, monitoring for splenomegaly [68] and bone mineral density assessment [69].
Long term safety	Available from the 20,000 annual healthy volunteer peripheral blood donors. Initial concerns of increased leukaemia risk alleviated by long term follow up [70].
Dose,	We took advice from haematology colleagues. G-CSF dosages ranged between single as required injections of 15million units to 30 million units twice weekly.
Administration	Pre-loaded syringe for subcutaneous injection, volume < 1 ml
Storage	Refrigerated
Cost	UK approx. £50 ($65) per dose
Institutional / systemic factors	High cost novel drug in psychiatric practice. Treating teams likely to be unfamiliar with usage and this may not be supported by all mental health organisations.

**Table 3:** Lithium and Neutrophils

Physiological effects	Enhances production of endogenous G-CSF, directly stimulating differentiation of stem cells and protecting neutrophils from the toxicity of some drugs, although to a far smaller extent than G-CSF.
Haematological usage	Reports of use to treat idiopathic neutropenia [71]
Lithium and clozapine rechallenge	Reviews by Manu (2012) [72] and Boazak (2019) [73] show high rates of success.
Adverse effects in clozapine rechallenge	Isolated case reports of failure / fatality [74] and of neurotoxicity [75]
Use, dose, side effects	Familiar to psychiatrists
Institutional / systemic factors	Systemic difficulties not likley

Many of our patients rechallenged with clozapine have had an excellent clinical response; significant numbers have now been transferred to conditions of lesser security. This creates a difficulty. Novel, expensive and high risk treatment strategies may not be endorsed at future placements. Several of our patients have had changes to treatment regimes in downstream settings. These have not been problematic.

## Data Availability

Not applicable.

## Abbreviations

ANC: Absolute neutrophil count
BEN: Benign ethnic neutropenia
CIA: Clozapine induced agranulocytosis
CIN: Clozapine induced neutropenia
FBC: Full blood count
G-CSF: Granulocyte colony stimulating factor
HLA: Human leukocyte antigen
